# Hand-assisted versus straight laparoscopy for colorectal surgery — a systematic review and meta-analysis

**DOI:** 10.1007/s00384-022-04272-x

**Published:** 2022-11-02

**Authors:** Ashley O. Frois, Yeqian Huang, Christopher J. Young

**Affiliations:** 1 The University of Sydney, Central Clinical School, Sydney, Australia; 2 Department of Colorectal Surgery, Royal Prince Alfred Hospital, Camperdown, NSW Australia; 3 Memorial Health System, 511 NE 10th Street Abilene, Abilene, KS 67410 USA

**Keywords:** Colorectal, Hand-assisted laparoscopic surgery, Meta-analysis

## Abstract

**Purpose:**

Hand-assisted laparoscopic surgery (HALS) is an alternative to straight laparoscopy (LAP) in colorectal surgery. Many studies have compared the two in terms of efficacy, complications, and outcomes. This meta-analysis aims to uncover if there are any significant differences in conversion rates, operative times, body mass index (BMI), incision lengths, intraoperative and postoperative complications, and length of stay.

**Methods:**

Comprehensive searches were performed on databases from their respective inceptions to 16 December 2021, with a manual search performed through Scopus. Randomized controlled trials (RCTs), cohort studies, and case series involving more than 10 patients were included.

**Results:**

A total of 47 studies were found fitting the inclusion criteria, with 5 RCTs, 41 cohort studies, and 1 case series. Hand-assisted laparoscopic surgery was associated with lower conversion rates (odds ratio [OR] 0.41, 95%CI 0.28–0.60, *p* < 0.00001), shorter operative times (Mean Difference [MD] − 8.32 min, 95%CI − 14.05– − 2.59, p = 0.004), and higher BMI (MD 0.79, 95%CI 0.46–1.13, *p* < 0.00001), but it was also associated with longer incision lengths (MD 2.19 cm, 95%CI 1.66–2.73 cm, *p* < 0.00001), and higher postoperative complication rates (OR 1.15, 95%CI 1.06–1.24, *p* = 0.0004). Length of stay was not different in HALS as compared to Lap (MD 0.16 days, 95%CI − 0.06–0.38 days, *p* = 0.16, and intraoperative complications were the same between both techniques.

**Conclusions:**

Hand-assisted laparoscopy is a suitable alternative to straight laparoscopy with benefits and risks. While there are many cohort studies comparing HALS and LAP, more RCTs would be needed for a better quality of evidence.

**Supplementary Information:**

The online version contains supplementary material available at 10.1007/s00384-022-04272-x.

## Background

The history of laparoscopic surgery spans back to the early 1900s, but only rapidly developed towards the end of the century [1]. As it matured, it offered an alternative to the traditional open surgeries, and provided benefits such as reduced intraoperative bleeding, postoperative pain, ileus, and hospital stay, at the expense of increased operative times [2]. 

Hand-assisted laparoscopic surgery (HALS), a variant of straight laparoscopic surgery, gained popularity in the mid-1990s with the introduction of hand-assisted devices [3]. It was seen as an alternative to straight laparoscopy (LAP) but with an easier learning curve, thus allowing an easier transition from open techniques to these minimally invasive methods [4].

Comparisons between HALS and LAP have been made in other surgical specialties [5], with the most recent reviews in the field of colorectal surgery published in 2008 by Aalbers [4] and a Cochrane Review by Moloo et al. [6] in 2010, which included 3 randomized controlled trials (RCTs). In the intervening years since, there have been many developments in operative techniques for both surgeries, in colorectal disease management, and consequently many new comparative studies have been performed.

This review aims to incorporate those studies in Aalbers’ and Moloo’s reports with the new advances published in the last decade. With the newly available data, it is important to provide clinicians with an updated, evidenced-based resource on which they can base their operative decisions.

## Methods

### Search strategy

An encompassing search with broad terms was performed on 16 December 2021 on the PreMEDLINE, MEDLINE, and Embase databases for articles related to HALS and LAP.

The following search protocol for MEDLINE is as follows:

1. hand assisted laparoscopic surgery.mp. or exp Hand-Assisted Laparoscopy/
2. hand port.mp.
3. 1 or 2
4. Laparoscopic Surgery.mp. or exp Laparoscopy/
5. 3 and 4

Keywords were changed if necessary, for the equivalents in the other databases. Reference searches were also performed using Scopus, and all articles were collated and reviewed.

## Study selection/inclusions and exclusions

Search results were screened independently by A.F. and Y.H. by their titles and abstracts according to a pre-determined set of inclusion and exclusion criteria, and disagreements were resolved by C.Y. In the interest of gathering as wide a dataset as possible, cohort studies and case series that had more than 10 patients and with quantifiable data were also included.

### Inclusion criteria

1. Studies that had a clearly documented data for population of HALS and LAP.
2. Studies with quantified outcomes of any one or more of the following:
  - conversion rate,
  - operative time,
  - body mass index (BMI),
  - length of incision,
  - complication rate, and,
  - length of stay.

### Exclusion criteria

1. Studies that involved other specialties of surgery (e.g., urology or upper gastrointestinal surgery),
2. In vitro models,
3. Non-quantitative studies such as opinion pieces or letters or the editor,
4. Conference abstracts, and,
5. Non-English texts

## Data extraction/data abstraction and analysis

Data from the included studies were extracted and compiled into tables comparing conversion rate, operative time, BMI, incision length, complication rate, and length of stay.

Conversion rate was defined as any deviation from the planned surgery, i.e., the data for LAP conversions includes cases that were converted into both HALS and open surgery.

Definitions of intraoperative and postoperative complications were flexible and varied between studies, hence all complication rates as reported and defined by the individual authors of each study were included. Only papers that clearly showed they were reporting their complication rates as any single complication happening per surgery were included. Papers that calculated their complication rates as a total number of complications among the number patients were excluded from this outcome measure analysis.

Data for continuous variables were collected as mean ± standard deviation (SD); however, in papers that reported a median and range, the formulae described in Hozo et al. [7] was used to estimate a mean ± SD for meta-analysis. For studies that reported their data as a median with an interquartile range, the formulae by Wan et al. [8] were used to estimate a mean ± SD.

## Assessments of methodological quality

The Cochrane Collaboration tool for assessing risk of bias [9] was used to assess the methodological quality of RCTS. Cohort studies were evaluated using the Newcastle–Ottawa Scale (NOS) [10]. The scale consists of 8 questions and has a maximum possible score of 9 stars. All articles were evaluated independently by A.F. and Y.H., and disagreements were put to C.Y. for mediation.

## Statistical analysis

All meta-analyses were performed using RevMan (version 5.3) [11], with a random effects analysis model used in all cases.

For continuous variables such as operative time, BMI, incision length, and length of stay, the mean difference (MD) was calculated using an inverse-variance (IV) statistical method and reported with 95% confidence intervals (95%CI). For dichotomous variables such as conversion rates and complication rates, an odds ratio (OR) was calculated using the Mantel–Haenszel statistical method and reported with 95%CI. A *p*-value of < 0.05 was predetermined to be the limit for statistical significance. An *I*^2^ value of greater than 60% was regarded as being heterogenous. Funnel plots were generated using RevMan to assess for publication bias.

Several papers reported their data as subgroups depending on disease or type of surgery (e.g., left hemicolectomies, right hemicolectomies). In these situations, an average mean and average standard deviation were calculated using the appropriate formulae.

RCTs were analyzed separately from the cohort studies/case studies. The MD or OR for each subgroup in each measure was reported individually, along with the overall value for the outcome.

## Results

### Selection results and characteristics of the studies

**Fig. 1:**
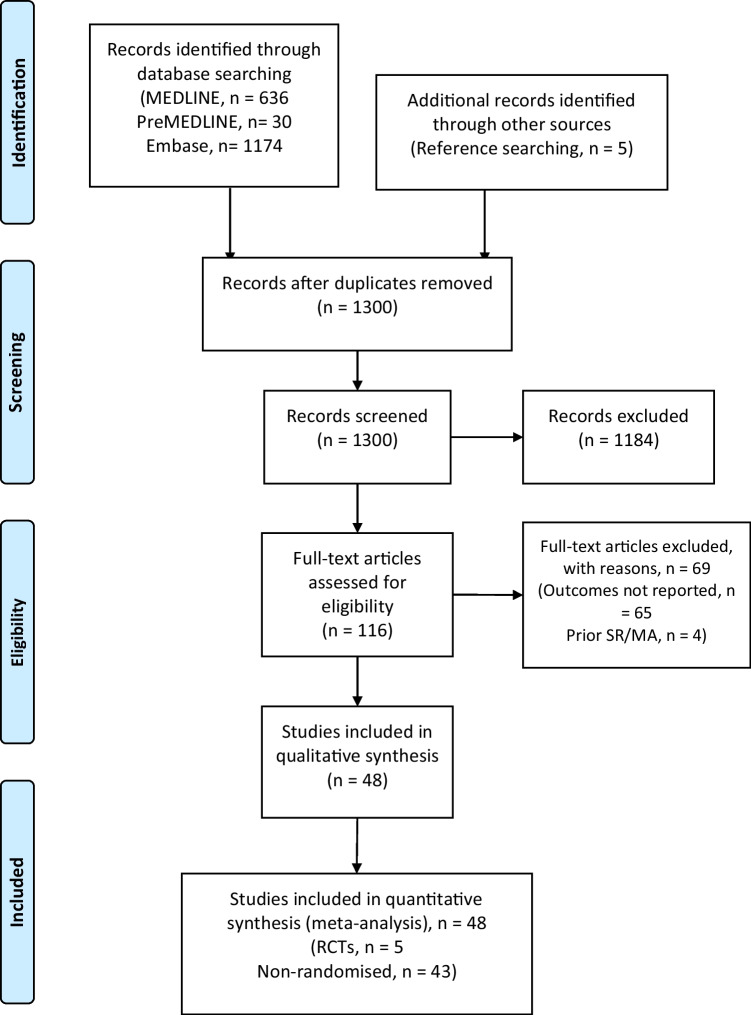
A total of 1604 records were identified and after removing duplicates and applying exclusion criteria, 44 articles remained

A total of 1352 records were obtained from the search after duplicates were removed. A further 1236 were removed after screening based on their title and abstract. The remaining 116 full-text articles were assessed for eligibility and 47 studies were included in this study [12–58]. A PRISMA flowchart was completed to illustrate this process (Fig. 1).

There were 5 RCTs [20, 27, 35, 48, 57] and 41 retrospective cohort studies[12–19, 21–26, 28–34, 36–47, 49–52, 54–56, 58], of which 8 were case-matched studies[17–19, 36, 38–40, 42, 58]. There was one case series [53] included in this review, as it had greater than 10 patients and data for each individual patient was recorded.

### Methodological quality

The 5 RCTs showed a low to moderate risk of bias. This is largely due to it being impossible to conduct a double-blind trial in this context (Table 1). In general, the 5 RCTs had low risk of bias in their randomization processes and in their reporting of the outcomes. The 42 cohort studies scored an average of 5.40 stars out of 9 on the NOS (Table 2).

### Publication bias

Funnel plots were generated with pseudo 95% confidence intervals [59, 60] for each outcome measured and are included in the appendix. In general, most measures showed an approximately symmetrical spread around the calculated mean difference or odds ratio, except for operative times which had more studies reporting a larger negative difference with a wider standard error (Supplementary figures, Fig. S1).

### The effects of interventions

#### Conversion rate

**Fig. 2:**
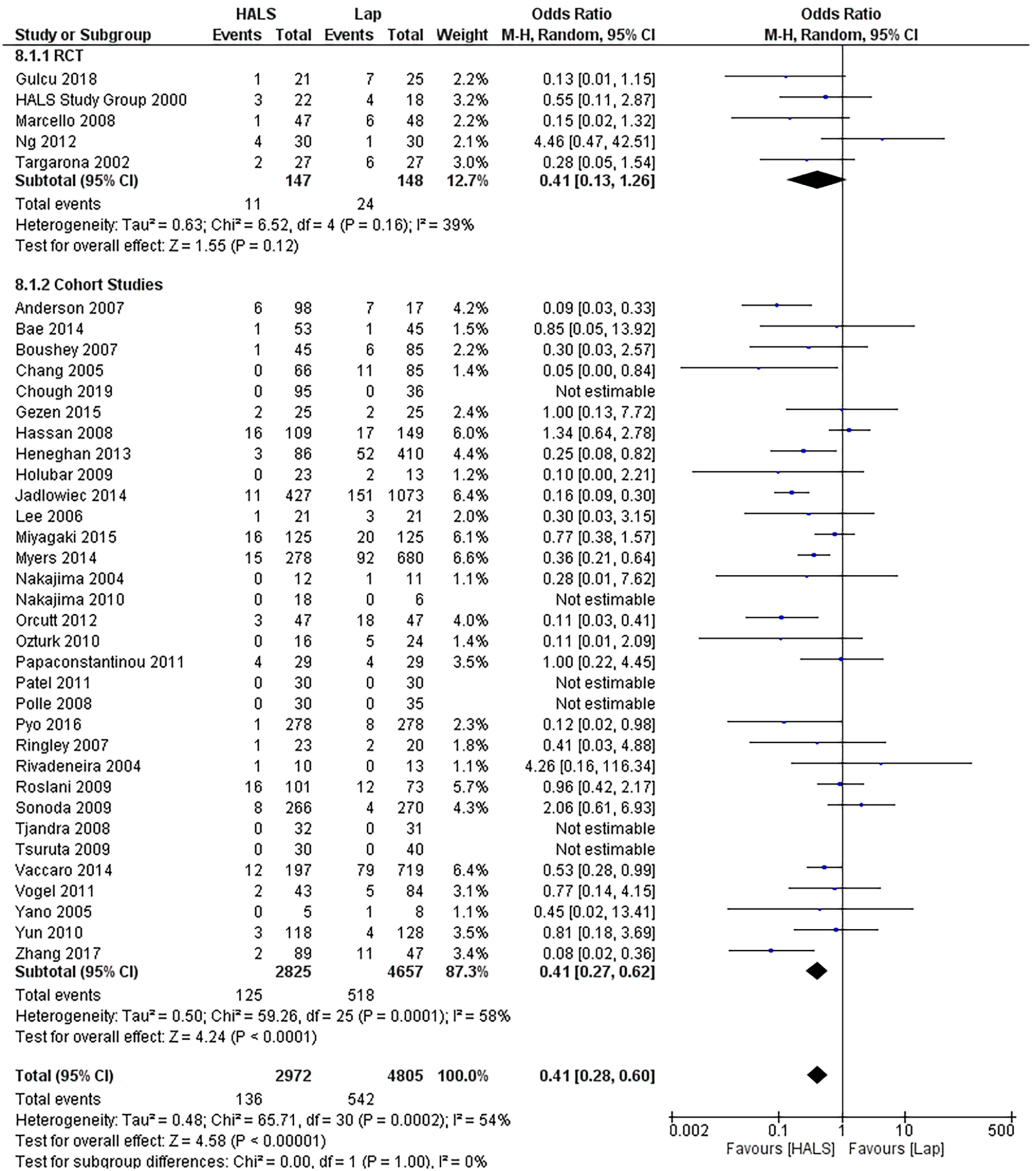
Forest plot of conversion rates in HALs and LAP. HALS had a lower converision rate (HALS, hand-assisted laparoscopy; LAP, straight laparaoscopy)

HALS was overall associated with a lower conversion rate than LAP, with an OR of 0.41 (95%CI 0.28–0.60, *p* < 0.0001) (Fig. 2). Six studies reported nil conversions in either arm [34, 40, 41, 49, 50, 56]. For the purpose of full reporting, they were included in the analysis despite not impacting the results. There were no common factors identifiable between the studies with no conversions, with varying patient population sizes and conditions ranging from diverticular disease to carcinoma. A subgroup analysis of only the RCTs showed the difference was not significant amongst the 5 RCTs (OR 0.41, 95%CI 0.13–1.26, *p* = 0.12).

#### Operative time

**Fig. 3:**
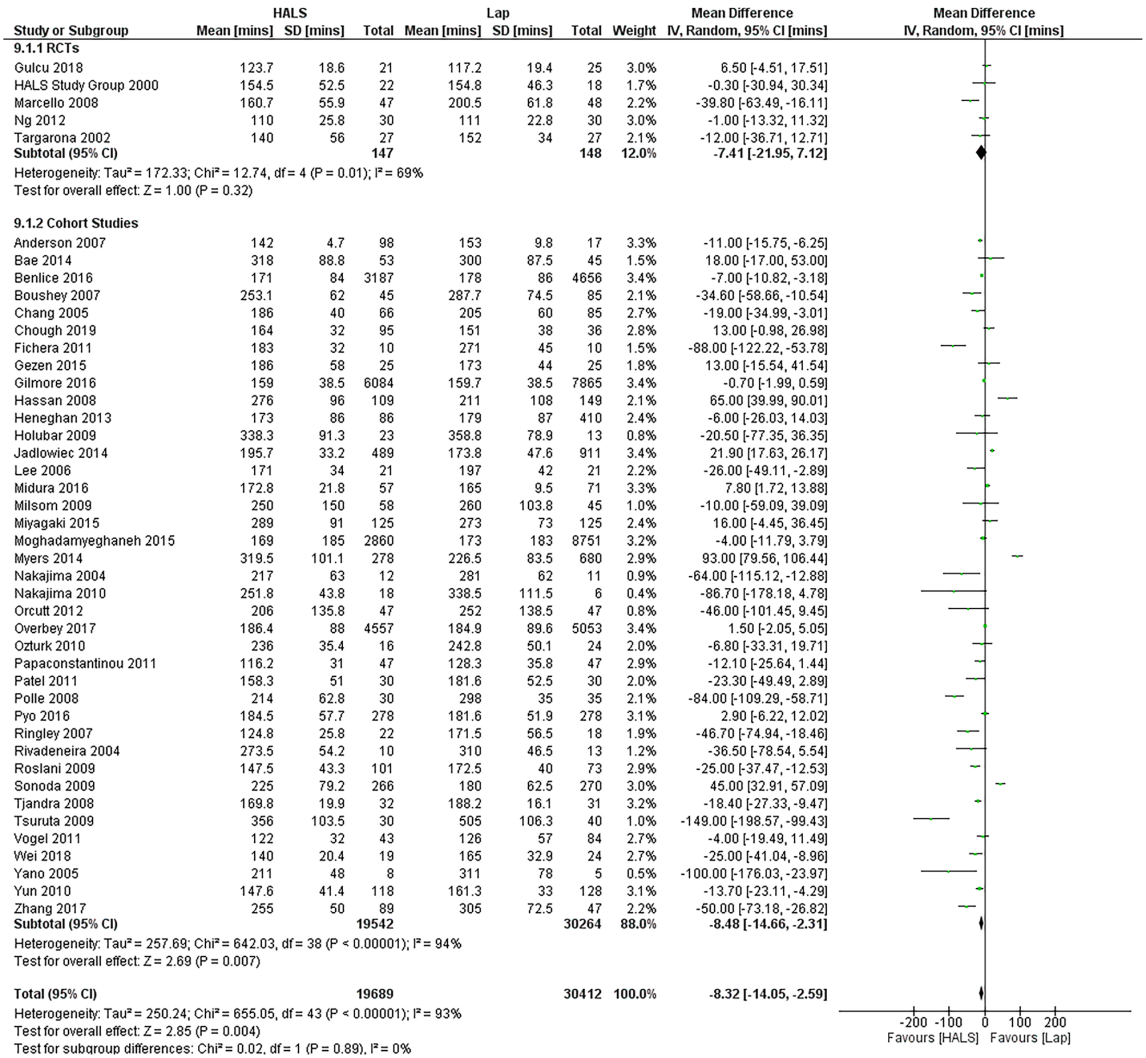
Forest plot of operative times in HALS and LAP. HALS was associated with shorter operative times (HALS, hand-assisted laparoscopy; LAP, straight laparaoscopy)

Operative times were found to be overall slightly shorter In HALS than in LAP (MD -8.32 min, 95%CI − 14.05– − 2.59, *p* = 0.004) (Fig. 3). A subgroup analysis of the RCTs showed there was no significant difference between HALS and LAP. The 5 studies that showed the greatest difference between HALS and LAP were cohort studies, two of which focused exclusively on restorative proctocolectomy [41, 50], one on total abdominal colectomy [17], one on both total and subtotal abdominal colectomies [34], and one on low anterior resections [53].

#### BMI

**Fig. 4:**
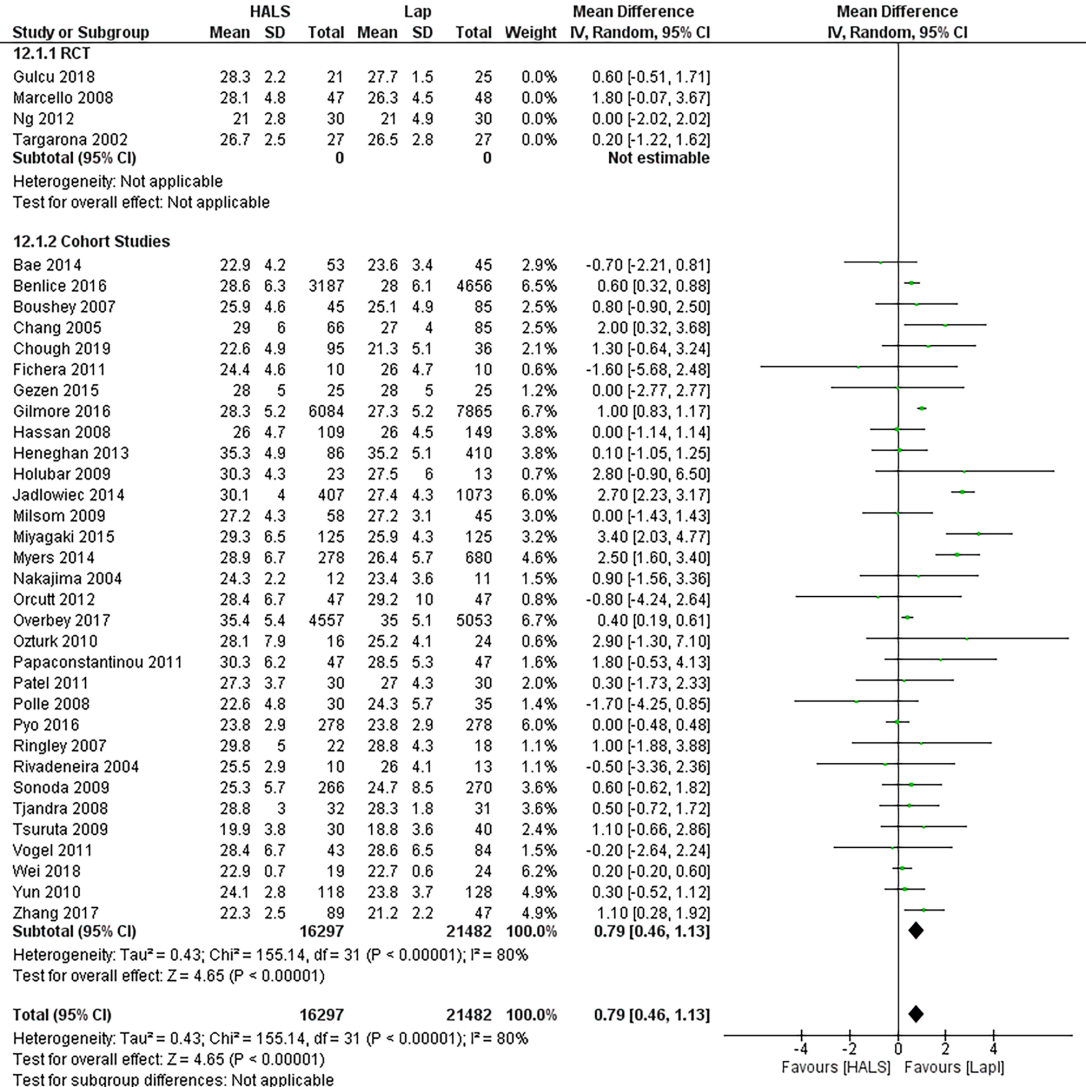
Forest plot of BMI as a potential contributing factor to surgeon choice of HALS or LAP. Patients who underwent LAP had a higher BMI in the studies included in this meta-analysis (HALS, hand-assisted laparoscopy; LAP, straight laparaoscopy)

BMI was analyzed as a pre-operative factor to see if it affected surgeon preference in choosing one operation over the other. HALS was overall associated with a higher BMI than LAP (MD 0.79, 95%CI 0.46–1.13, *p* < 0.00001) (Fig. 4). The study with the most significant MD of 3.40 [2.03, 4.77] was a retrospective cohort study focusing purely on diverticulitis [30], and it was noted that HALS was more often used in complex cases. The RCTs were not included in this pre-operative factor measure as the groups are randomized pre-operatively.

#### Incision length

Incision lengths were longer in HALS as compared to LAP (MD 2.19 cm, 95%CI 1.66–2.73 cm, *p* < 0.00001) (Fig. S2). All studies that had reported incision lengths were unanimous in reporting a longer mean incision length in HALS as compared to LAP; however, a subgroup analysis of the RCTs showed no significant difference (MD 1.04 cm, 95%CI − 0.52–2.61, *p* = 0.19). It should be noted that only 2 of the 5 RCTs reported incision length.

#### Complication rates

There was no significant difference in intraoperative complication rates (OR 1.13, 95%CI 0.59–2.20, *p* = 0.71) between HALS and LAP (Fig. S3). HALS was, however, associated with a higher postoperative complication rate than LAP (OR 1.15, 95%CI 1.06–1.24, *p* = 0.0004) (Fig. S4). Similarly, as with the other outcome measures, the RCTs did not show a significant difference between HALS or LAP in terms of postoperative complication rates.

#### Length of stay

Hospital stay was not significantly different between HALS and LAP (MD 0.16 days, 95%CI **-**0.06–0.38 days, *p* = 0.16) (Fig. S5). None of the included studies found any significant difference between HALS and LAP in length of stay.

#### Differences between malignant and non-malignant diseases

A separate subgroup analysis was performed by grouping studies on whether they exclusively included patients with malignant disease or non-malignant diseases (which includes diverticulitis, inflammatory diseases, and slow bowel transit). There was a statistically significant reduction in conversion rates in malignant disease but not so in non-malignant disease, although the effect sizes were similar (OR 0.37, *p* = 0.03 VS OR 0.30, *p* = 0.07 respectively). Interestingly, while there was a statistically significant reduction in the operative times for both malignant and non-malignant diseases, non-malignant cases appeared to have a significantly larger effect size (MD − 15.05 min vs MD **-**55.63 min respectively). There were no remarkable differences between incision length, length of stay, complication rates, and BMI when comparing malignant disease to non-malignant diseases (Fig. S6-S12).

## Discussion

This review which incorporates 5 RCTs, 41 retrospective cohort studies, and 1 case series, shows that HALS has some advantages and disadvantages compared to LAP. The benefits of a lowered conversion rate and shortened operative times comes at the cost of an increased incision length and postoperative complication rates.

Operative times were found to be reduced in HALS, which is in line with the current literature that reports that HALS generally provides a reduced learning curve [38]. The tactile feedback helps the surgeon in spatial orientation and allows for a more efficient surgery. This is in line with the finding of a decreased conversion rate in HALS, as it allows for increased maneuverability in the abdominal cavity. One might expect a lower conversion rate to be associated with lowered intraoperative complication rates, but no significant difference between HALS and LAP was found for this outcome measure in our study.

Multiple studies have looked at HALS and LAP in the setting of obese patients, finding that HALS was associated with lower conversion rates in patients with high BMI [22]. The results of our study suggests that BMI may have been a factor in determining if a patient was planned for a HALS or a LAP in the included retrospective studies.

Incision lengths were found to be longer in HALS than in LAP, which is an expected result, as a longer incision is necessary to accommodate the hand port for the surgeon. The mean increase in incision length of 2.19 cm may also not be a clinically important result. Interestingly, the 2 RCTS [20, 27] that reported this outcome measure did not show significant difference in incision lengths between HALS and LAP when a subgroup analysis was performed.

HALS was however associated with an increased postoperative complication rate as compared to LAP surgeries. Similar to the previous point on BMI, this may perhaps be secondary to surgeon preference for HALS in more complicated surgical cases, hence predisposing HALS to more postoperative complications. Unfortunately, as most of the studies included in review are retrospective and non-randomized, this is a factor that cannot be accounted for. Nonetheless, this increased postoperative complication rate is not associated with a longer length of hospital stay, as the difference between HALS and LAP of 0.16 days (95%CI 0.06–0.38) was not statistically significant.

One consistent trend across the various outcome measures is that the RCTs did not show any significant difference between HALS and LAP when a subgroup analysis was performed. This could perhaps indicate that the perceived benefits and drawbacks are arising from inherent biases in the cohort studies (and the one case study). However, it should be noted that there are only 5 RCTs that fit our criteria, with 90% of our studies being non-randomized trials.

A strength of this study is its comprehensiveness — including any study that involves colorectal surgery, HALS, LAP, and fitting the rigorous selection criteria. The objective of this review is to study HALS and LAP as used in colorectal surgery, and not have it limited to particular techniques or conditions. However, this may have had an effect on diluting the results. It is possible that the above results between HALS and laparoscopy might differ depending on the procedure being performed. Similarly, the pathology in question might also influence the outcomes analyzed in this paper. More detailed reviews of the subgroups of malignant disease, inflammatory conditions, infective conditions, or emergency versus elective procedures, might yield some interesting results, but these are unfortunately beyond the scope of this paper.

On a similar note, a source of bias in this review is that most of the included studies are non-randomized. Several studies have noted within their cohorts that HALS was used more frequently in complex cases [13, 24], with Miyagaki et al. [30] finding in their study that HALS was used in more complex diverticular disease cases and was consequently associated with a higher postoperative complication rate and a longer length of stay.

Publication bias, on the other hand, is likely to be low in this review. Funnel plots for each outcome measure can be found in the supplementary figures (Fig. S1). Conversion rates, BMI, incision lengths, intraoperative and postoperative complication rates, and length of stay showed approximately symmetrical graphs, implying a lower risk of publication bias. In the study of operative times, however, the graph is slightly skewed towards having more publications that favor a shorter operating time for HALS, as well as more studies with a lower standard error in their reported mean difference in operative times.

Ultimately, there is a wealth of evidence included in this study, but more research will be required if a higher quality of evidence is desired. Perhaps one direction future studies could take would be to look at HALS versus LAP in specific patient subpopulations and for specific procedures, which can provide more a more granular guide for clinicians in decision-making. RCTs would ideally be the study of choice to reduce bias from preoperative factors influencing surgeon decisions for which procedure to perform.

## Conclusion

HALS is a viable alternative to LAP for colorectal surgery. The decision for choosing one or the other depends on the weighing up the benefits of lower conversion rates and shorter operative times, with the risks of longer incision lengths and higher postoperative complication rates. There are possible sources of bias in the studies included in this review, and in the review itself, which may understate some results, and more RCTs need to be performed for a higher level of evidence.

**Table 1:**
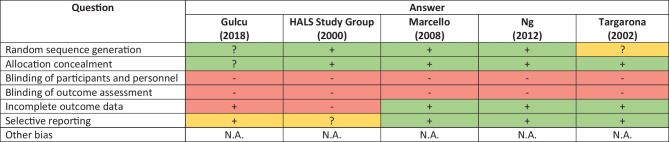
Green boxes with a ‘ + ’ indicates a perceived low risk of bias, while red boxes with a “-” indicates a perceived high risk of bias in the relevant category. Yellow boxes with a ‘?’ indicates an unclear risk of bias

**Table 2:**
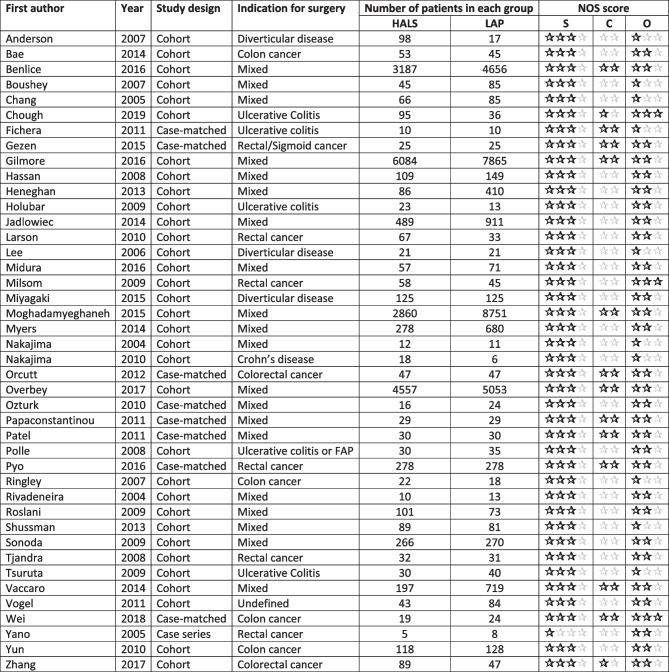
List of articles included in the study and their properties. Newcastle-Ottawa Scale reported as score for selection (**S**), comparability (**C**), and outcome (**O**)

## Supplementary Information

Below is the link to the electronic supplementary material.

Supplementary file1 (DOCX 594 KB)
