# Supplementary material for: Hand-assisted versus straight laparoscopy for colorectal surgery — a systematic review and meta-analysis
Source: Int J Colorectal Dis. 2022 Nov 2;37(11):2309–19. doi: 10.1007/s00384-022-04272-x (PMC9640416; doi:10.1007/s00384-022-04272-x)
Supplement: Supplementary file 1 — Supplementary file1 (DOCX 594 KB) [file 384_2022_4272_MOESM1_ESM.docx]

# Supplementary Figures


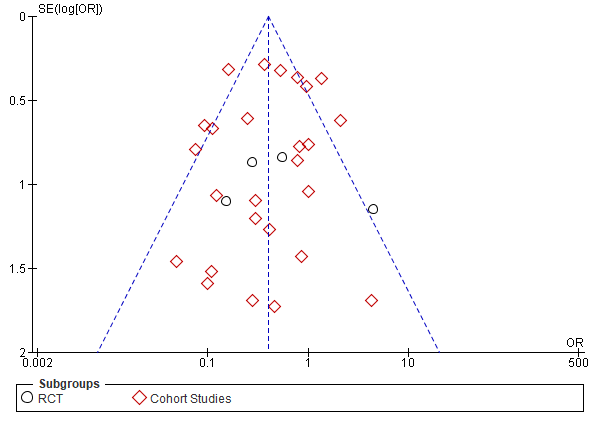

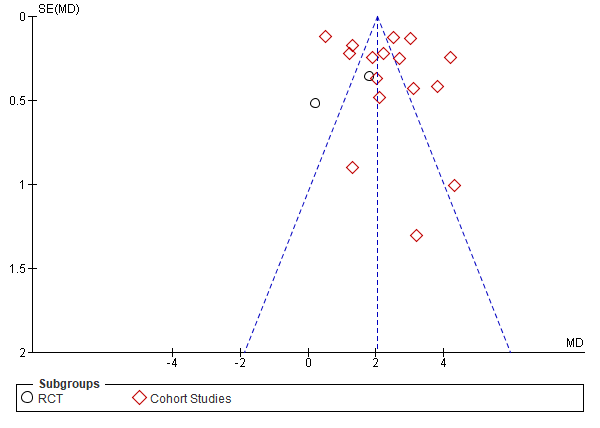

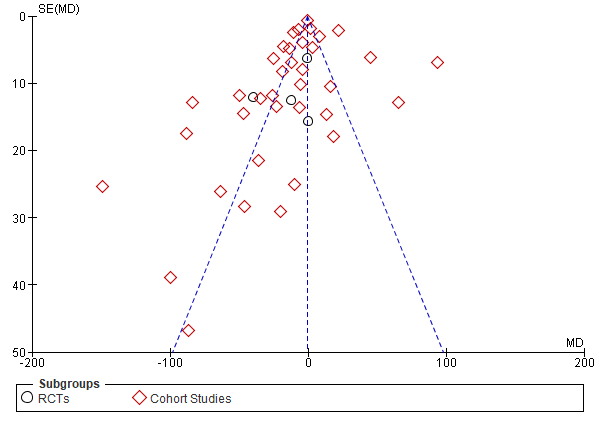

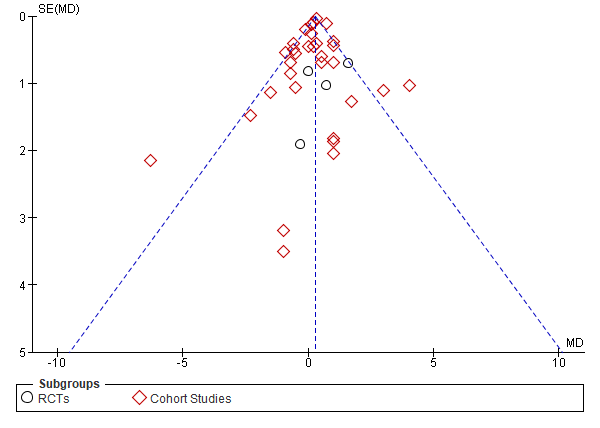

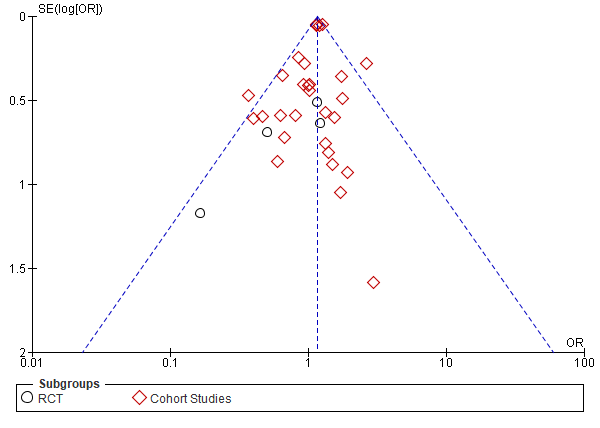

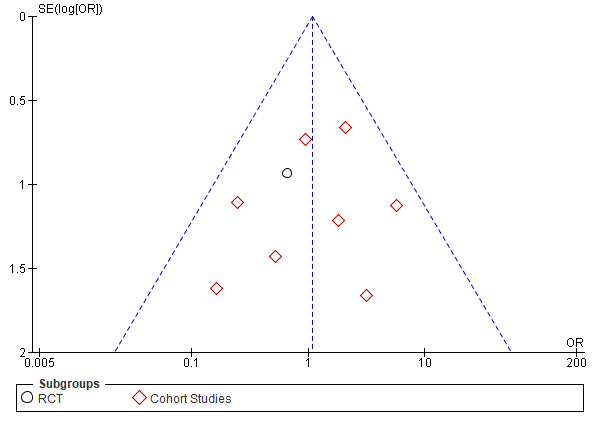

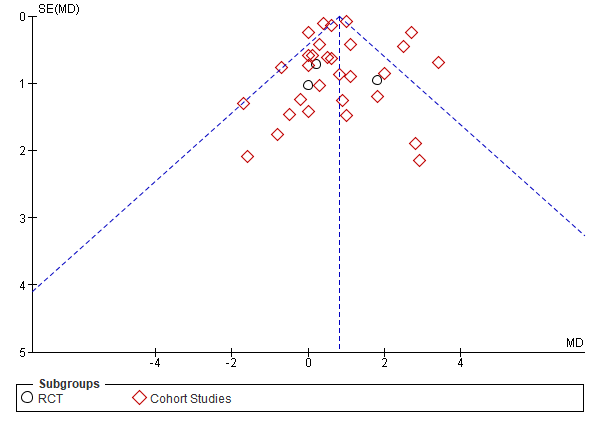


**a**

**b**

**c**

**d**

**e**

**f**

**g**

**Fig.** **S1** Funnel plots with pseudo 95%CI intervals showing no publication bias in the measured outcomes. **a** Conversion rate; **b** Operative time; **c** BMI; **d** Incision length; **e** Postoperative complication rate; **f** Intraoperative complication rate; **g** Length of stay


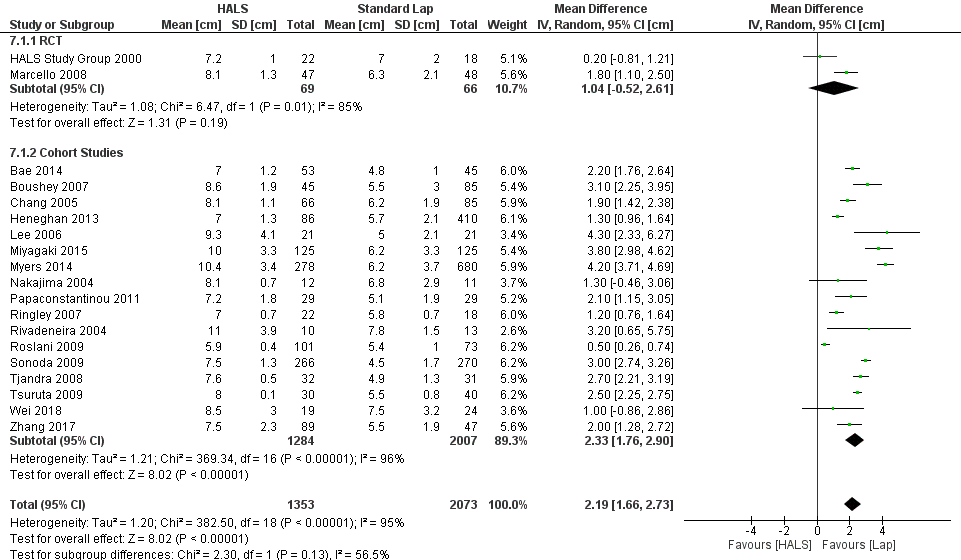


**Fig.** **S2** Forest plot of incision lengths of HALS as compared to LAP. Incision lengths were longer in HALs. (HALS: Hand-assisted laparoscopy; LAP: Straight laparaoscopy)


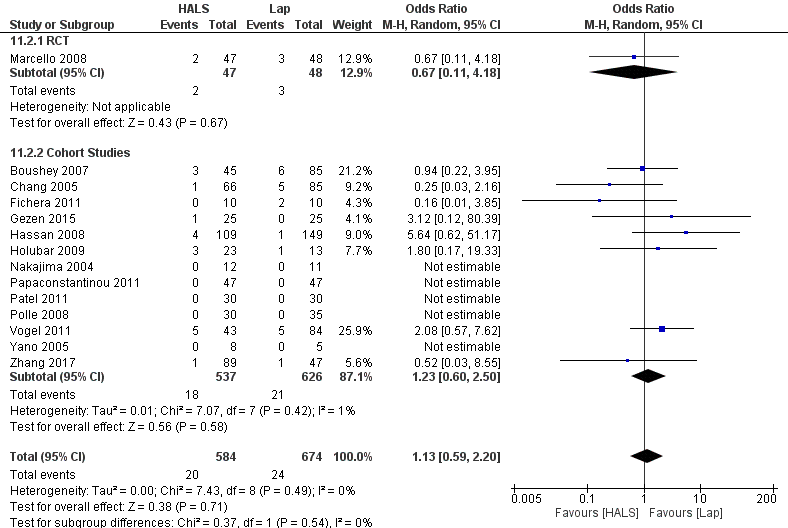


**Fig.** **S3** Intra-operative complication rates in HALS and LAP. There was no significant difference between the two procedures. (HALS: Hand-assisted laparoscopy; LAP: Straight laparaoscopy)


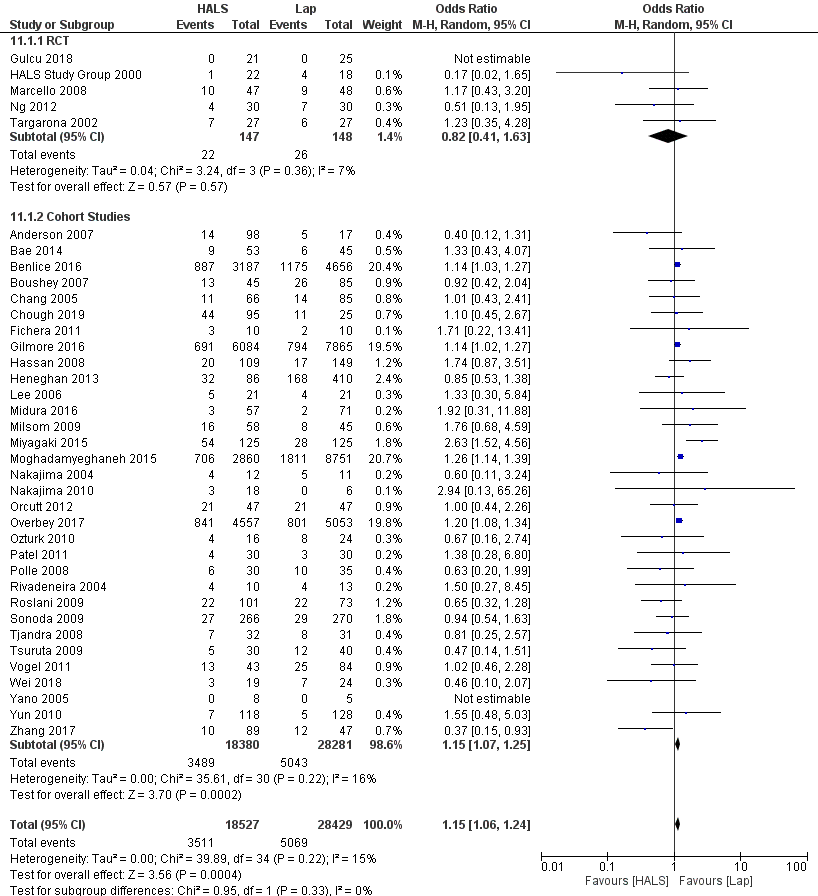


**Fig.** **S4** Postoperative complication rates in HALS and LAP. HALS had a higher incidence of postoperative complications. (HALS: Hand-assisted laparoscopy; LAP: Straight laparaoscopy)


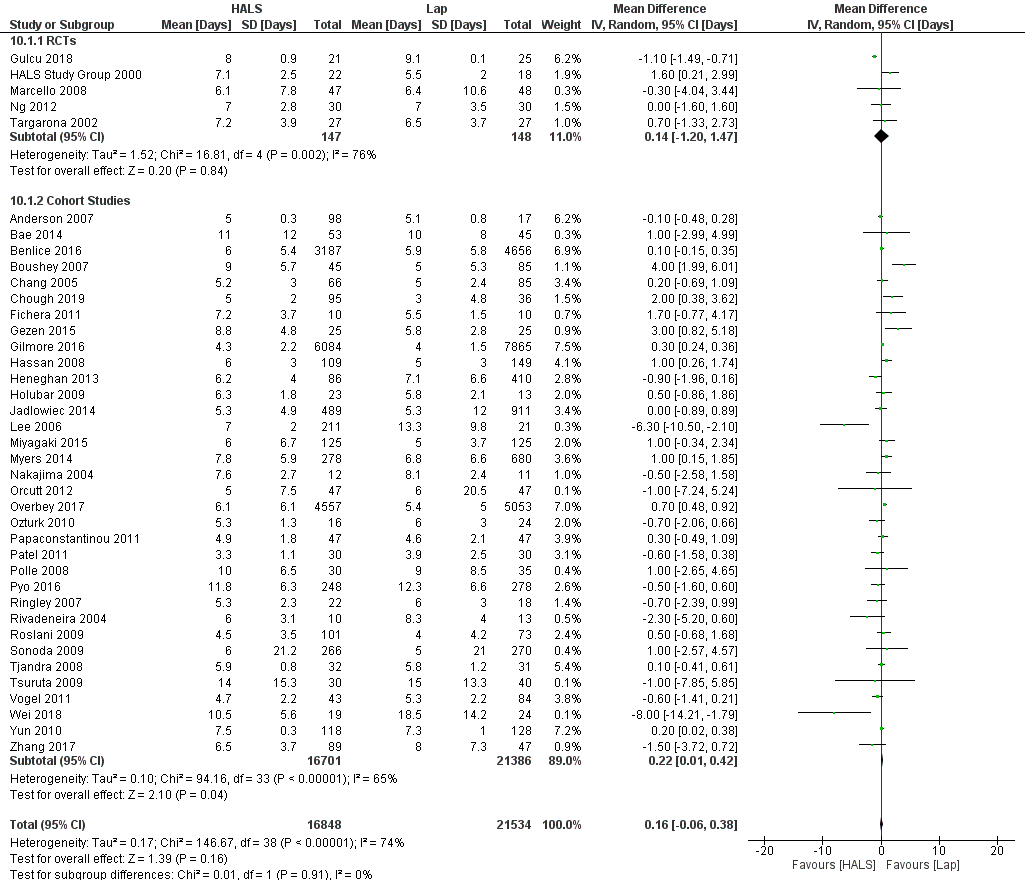


**Fig.** **S5** Length of stay in HALS and LAP. HALS was associated with a longer length of stay than LAP. (HALS: Hand-assisted laparoscopy; LAP: Straight laparaoscopy)


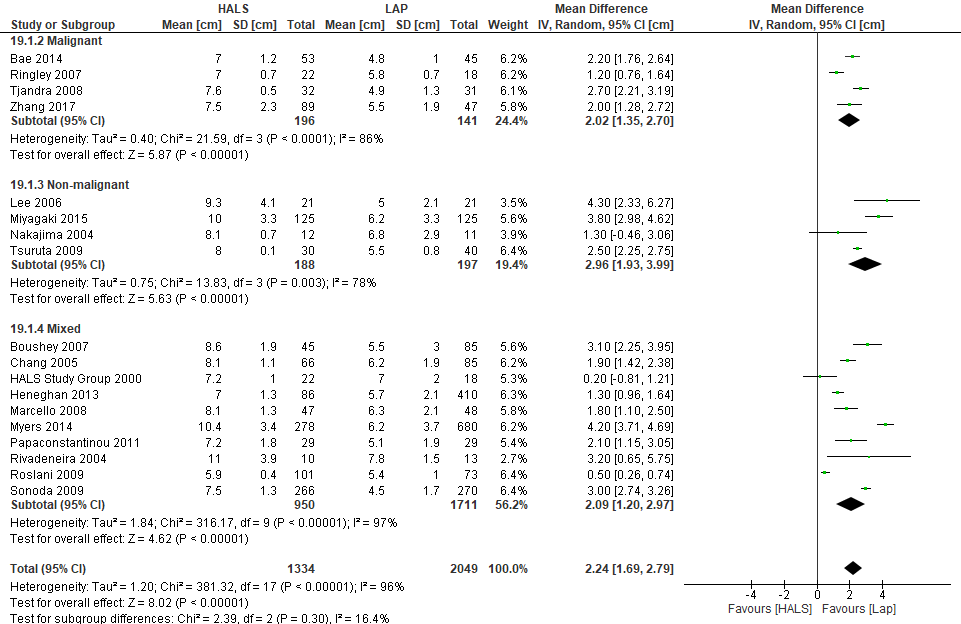


**Fig. S6**

Incision length in HALS and LAP, studies divided into subgroups of malignant disease only, non-malignant disease only, and having both malignant and non-malignant disease.

**
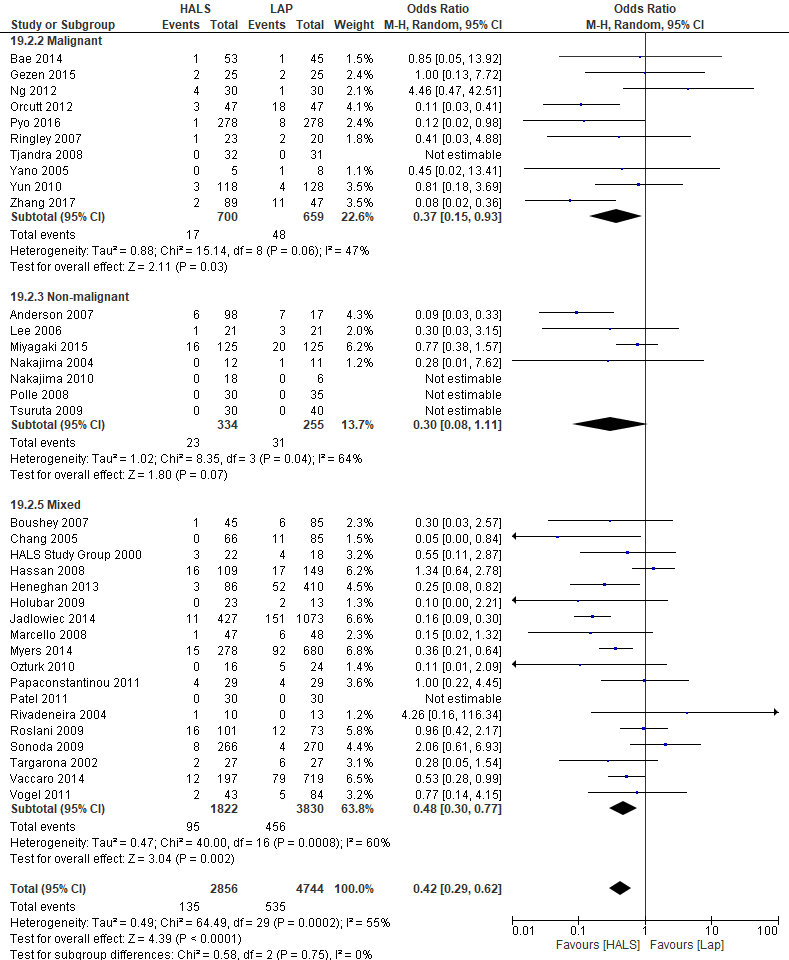
**

**Fig. S7**

Conversion rates in HALS and LAP, studies divided into subgroups of malignant disease only, non-malignant disease only, and having both malignant and non-malignant disease.

**
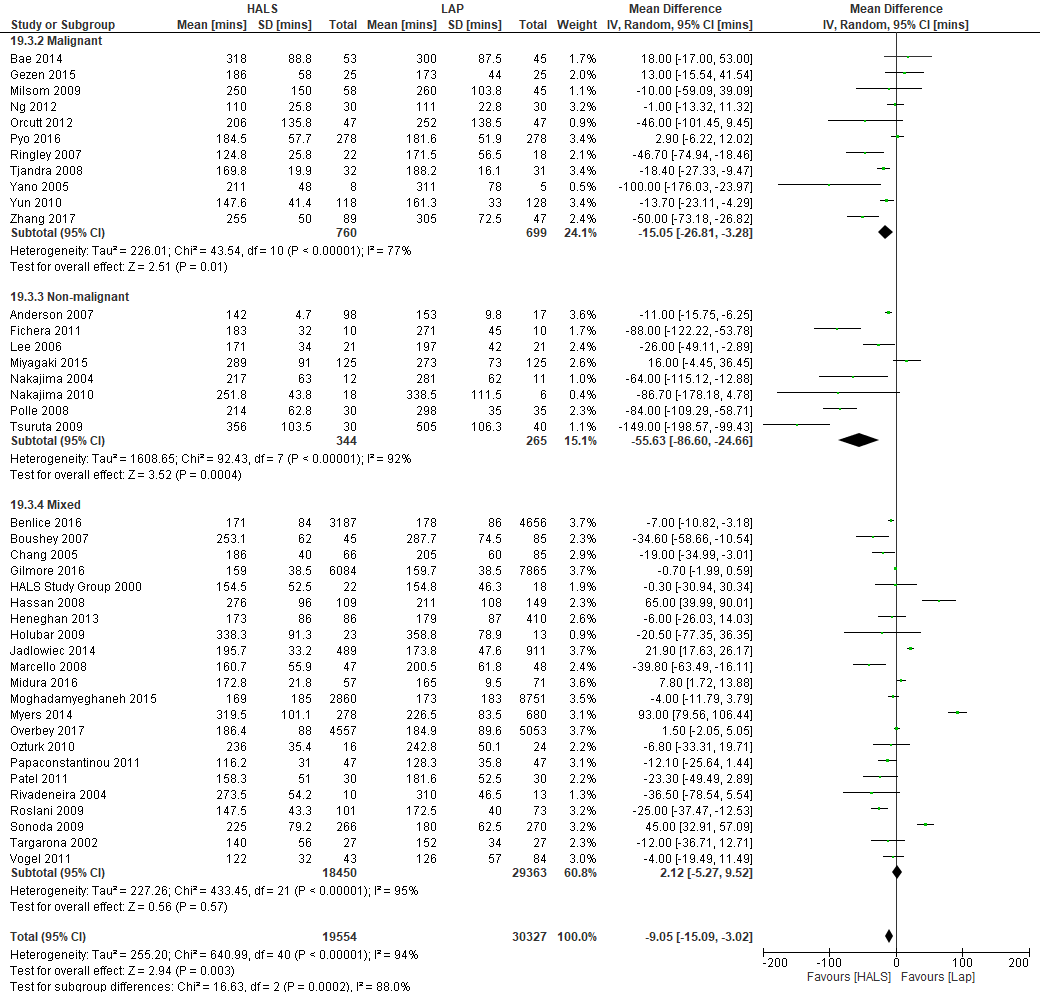
**

**Fig. S8**

Operative time in HALS and LAP, studies divided into subgroups of malignant disease only, non-malignant disease only, and having both malignant and non-malignant disease.

**
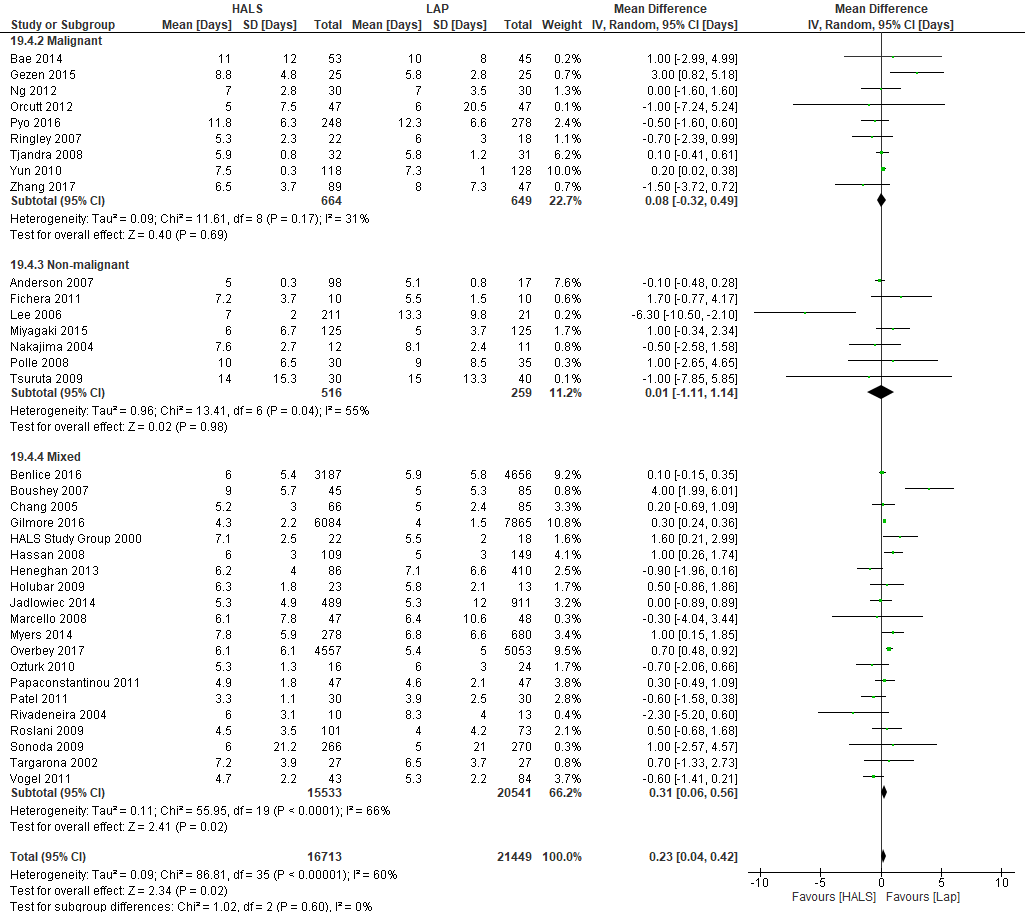
**

**Fig. S9**

Length of stay in HALS and LAP, studies divided into subgroups of malignant disease only, non-malignant disease only, and having both malignant and non-malignant disease.

**
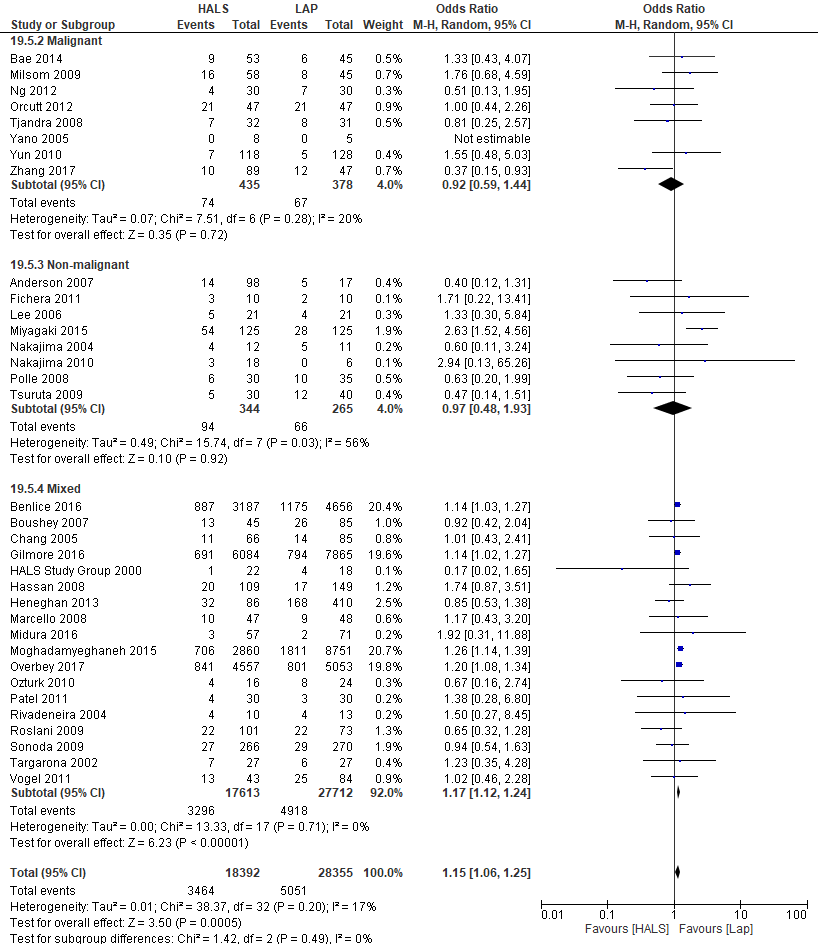
**

**Fig. S10**

Postoperative complication rates in HALS and LAP, studies divided into subgroups of malignant disease only, non-malignant disease only, and having both malignant and non-malignant disease.

**
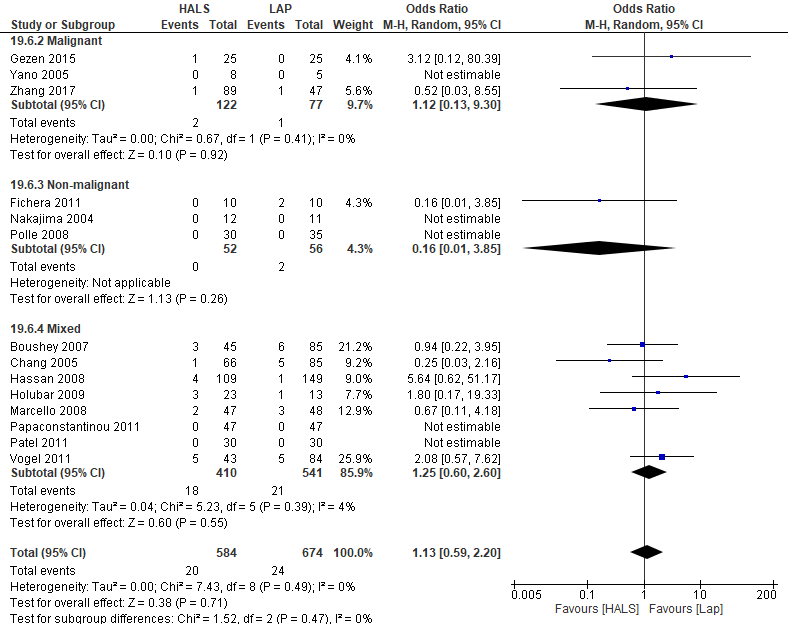
**

**Fig. S11**

Intraoperative complication rates in HALS and LAP, studies divided into subgroups of malignant disease only, non-malignant disease only, and having both malignant and non-malignant disease.

**
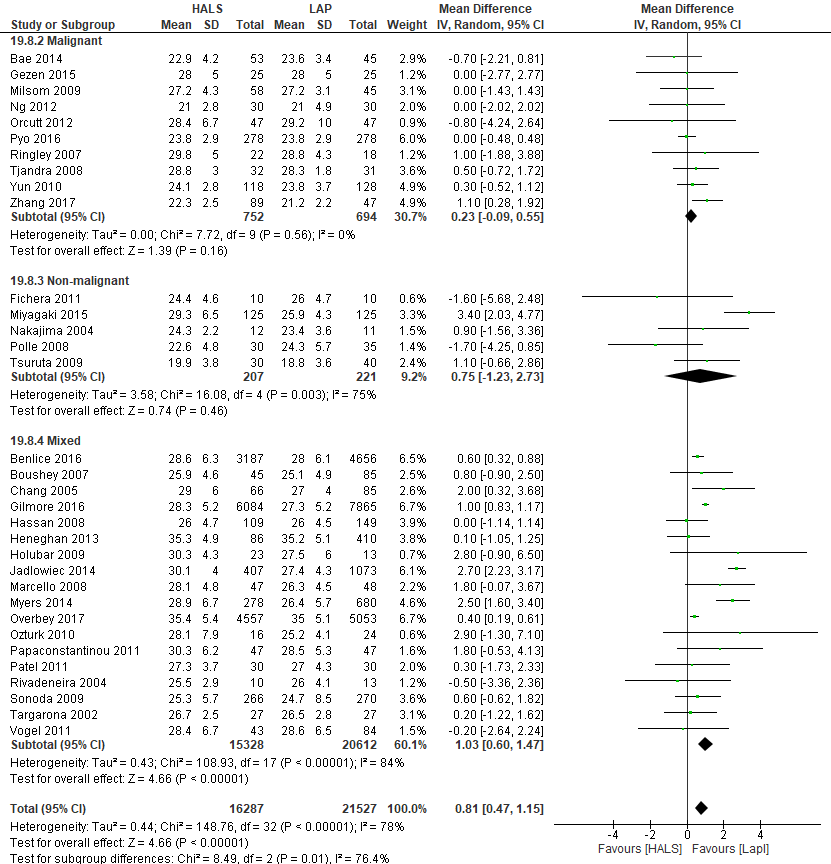
**

**Fig. S12**

BMI in HALS and LAP, studies divided into subgroups of malignant disease only, non-malignant disease only, and having both malignant and non-malignant disease.
